# Evaluation of normalization methods for two-channel microRNA microarrays

**DOI:** 10.1186/1479-5876-8-69

**Published:** 2010-07-21

**Authors:** Yingdong Zhao, Ena Wang, Hui Liu, Melissa Rotunno, Jill Koshiol, Francesco M Marincola, Maria Teresa Landi, Lisa M McShane

**Affiliations:** 1 Division of Cancer Treatment and Diagnosis, National Cancer Institute, National Institutes of Health, Bethesda, Maryland, USA; 2 Department of Transfusion Medicine, Clinical Center, National Institutes of Health, Bethesda, Maryland, USA; 3 Division of Cancer Epidemiology and Genetics, National Cancer Institute, National Institutes of Health, Bethesda, Maryland, USA

## Abstract

**Background:**

MiR arrays distinguish themselves from gene expression arrays by their more limited number of probes, and the shorter and less flexible sequence in probe design. Robust data processing and analysis methods tailored to the unique characteristics of miR arrays are greatly needed. Assumptions underlying commonly used normalization methods for gene expression microarrays containing tens of thousands or more probes may not hold for miR microarrays. Findings from previous studies have sometimes been inconclusive or contradictory. Further studies to determine optimal normalization methods for miR microarrays are needed.

**Methods:**

We evaluated many different normalization methods for data generated with a custom-made two channel miR microarray using two data sets that have technical replicates from several different cell lines. The impact of each normalization method was examined on both within miR error variance (between replicate arrays) and between miR variance to determine which normalization methods minimized differences between replicate samples while preserving differences between biologically distinct miRs.

**Results:**

Lowess normalization generally did not perform as well as the other methods, and quantile normalization based on an invariant set showed the best performance in many cases unless restricted to a very small invariant set. Global median and global mean methods performed reasonably well in both data sets and have the advantage of computational simplicity.

**Conclusions:**

Researchers need to consider carefully which assumptions underlying the different normalization methods appear most reasonable for their experimental setting and possibly consider more than one normalization approach to determine the sensitivity of their results to normalization method used.

## Background

MicroRNAs (miRs) are a class of short, highly conserved non-coding RNAs known to play important roles in numerous developmental processes. MiRs regulate gene expression through incomplete base-pairing to a complementary sequence in the 3' untranslated region (3' UTR) of a target mRNA, resulting in translational repression and, to a lesser extent, accelerated turnover of the target transcript [1]. Recently, the dysregulation of miRs has been linked to cancer initiation and progression [2], indicating that miRs may play roles as tumor suppressor genes or oncogenes [3]. There is also mounting evidence that miRs are important in development timing [4,5], cell differentiation [6], cell cycle control and apoptosis [7]. The involvement of miRs in those biological functions suggests their intrinsic roles in maintaining homeostasis or contributing to pathological processes.

Technologies utilized for relative quantification of miR expression include Northern blot, real time PCR, in situ hybridization, sequence analysis and array-based profiling [8]. Due to the limited throughput of other technologies, microarray-based miR profiling has become a popular method for interrogation of miRs, especially when the contributions of specific miRs to a given condition or process remain elusive. However, miR arrays distinguish themselves from gene expression arrays by their more limited number of probes, and the shorter and less flexible sequence in probe design. Robust data processing and analysis methods tailored to the unique characteristics of miR arrays are greatly needed.

Normalization is a key early step in miR microarray data processing. Normalization methods are aimed at removing data artifacts resulting from systematic or random technical variation. If not removed, these artifacts might affect subsequent data analyses, such as class comparison and class prediction. Assumptions underlying commonly used normalization methods for gene expression microarrays containing tens of thousands or more probes may not hold for miR microarrays. Further studies to determine optimal normalization methods for miR microarrays are needed. The best normalization method may differ depending on whether the miR chip uses a one-channel or two-channel system. In a one channel system, single samples are labeled and hybridized to individual arrays. For arrays using a two-channel system, generally two samples are separately labeled, mixed, and hybridized together to each array. The most commonly used design for a two-channel system is called the reference design. One of the samples is used as an internal standard so that the signal intensity which reflects the amount of hybridization to a probe for a sample of interest is measured relative to the intensity for the same probe on the same array for the reference sample [9].

Several papers comparing miR microarray normalization methods have been published; however, the results and recommendations are not consistent. Rao et al [10] compared normalization methods for single channel miR microarray data. They reported that quantile normalization was the best performing method for reducing the differences in microRNA expression values among replicate tissue samples. Pradervand et al. [11] confirmed that quantile normalization was the most robust normalization method for their set of invariant miRs using the Agilent single channel platform. In contrast, Hua et al. [12], using Rt-PCR as a gold standard, found that the lowess method gave the best result for two-channel miR microarray data, although the differences among their top performing methods were minimal. However, the suitability of Rt-PCR as a comparator for miR microarray expression results has been questioned [8,13], and the stability of lowess smoothers is known to be dependent on the number of data points to which they are applied. Sarkar et al. [14] reported quality assessment for two- channel miR expression arrays, and they found that all normalization methods performed adequately in their study.

Here we report our evaluation of many different normalization methods on a custom-made two channel miR microarray. Our study examined technical replicates from a large number of different cell lines to determine which normalization methods minimized differences between replicate samples while preserving differences between biologically distinct miRs.

## Methods

### Cell line culture

Ten lung carcinoma cell lines from the NCI60 panel were obtained from the National Cancer Institute's Developmental Therapeutics Program (DTP), and 9 renal cell carcinoma cell lines were generated at the Surgery Branch, National Cancer Institute, National Institutes of Health (NIH). All cell lines were cultured in complete RPMI media supplemented with 10% FBS, 1 mM HEPES, 1 mM Ciprofloxacin and L-glutamine/penicillin/streptomycin. All cells were cultured at 37°C under 5% CO_2_. Cells were harvested at sub-confluent condition by trypsin-versene (Invitrogen) detachment and centrifugation after 3-5 days in culture. A single EBV cell line used as the reference sample was cultured in the same media in suspensional growth cells and harvested by centrifugation at 1200 rpm for 5 min after one week of culture. Cell pellets were immediately lysed in Trizol at 1-2e7 cell per ml of Trizol.

### RNA isolation and labeling

Total RNA from 10 lung carcinoma cell lines and 9 renal cell carcinoma cell lines were isolated using Trizol reagent. Small RNA in total RNA samples were enriched and purified by flashPAGE Fractionator (Ambion, Austin, TX USA) according to the manufacturer's instruction. The reference sample consisting of one EBV cell line was processed following identical procedures. After small RNA purification, small RNA from test samples and EBV reference samples, equivalent to 10 μg of the total RNA, were labeled with Cy5 and Cy3, respectively, using *mir*Vana™ miRNA Labeling Kit (Ambion, Austin, TX USA).

### Microarray fabrication and quality control procedures

A custom-made oligo array including 714 human, mammalian and viral mature antisense miRs (mirbase: http://microrna.sanger.ac.uk/, version9.1) plus 2 internal controls with 7 serial dilutions [2,6,15] were printed at Infectious Disease and Immunogenetics Section, Department of Transfusion Medicine, Clinical Center, NIH. The antisense miR oligo probes were 5' amine modified and immobilized in duplicate (two spots per miR per array) on CodeLink activated slides (GE Health, NJ, USA) via covalent binding. Serially diluted control probes were used as indicators of labeling efficiency, optimization of intensity saturation, and intensity balance of test *vs*. reference sample. A single large labeling reaction of the EBV reference samples was used for all arrays. Strong and positive EBV-miR hybridization also functioned as a positive control quality assessment of the reference sample.

### Sample hybridization and image analysis

Equal amounts of labeled test and reference samples were cohybridized on the custom made miR oligo microarray for more than 14 hours at room temperature. After washing, the array was scanned using a GenePix 4B scanner. Any spot smaller than 25 pixels was filtered out and excluded from remaining analyses. If both channels produced intensities less than 100 for a given microRNA, that spot was also filtered out. For spots with one channel intensity less than 100 but the other channel intensity 100 or greater, the signal less than 100 was set to 100 prior to calculation of the signal ratio. The intensity ratio for each spot was then calculated as the red signal intensity (test samples) divided by the green channel signal intensity (EBV reference samples). Both single channel intensities and intensity ratios were log transformed (base 2) for normalization and further analyses. Overall, 9 out of 10 lung carcinoma cell lines and all 9 renal cell carcinoma cell lines have duplicate samples while one lung carcinoma cell lines has quadruplicate samples.

### Normalization Methods

1) Median

This normalization method uses the global median of log intensity ratios on each chip as the normalization factor. The global median log intensity ratio is calculated across all spots on the chip, and then this value is subtracted from the log intensity ratio for each spot. The global median of the normalized log intensity ratios equals zero.

2) Mean

This normalization method uses the global mean of log intensity ratios on each chip as the normalization factor. The global mean log intensity ratio is calculated across all spots on the chip, and then this value is subtracted from the log intensity ratio for each spot. The global mean of the normalized log intensity ratios equals zero.

3) Trimmed Mean

This normalization method is similar to the mean normalization method except that a trimmed mean of log intensity ratios on each chip is used as the normalization factor in place of the overall mean. A trimmed mean is calculated by discarding a certain percentage of the lowest and the highest log intensity ratios and then computing the mean of the remaining log intensity values. It is less susceptible to the effects of extreme values. In our experiments, we used a trimming percentage of 1% from both the lowest and highest data values.

4) Lowess

Lowess normalization assumes that the dye bias might be dependent on spot intensity. Let (*logG, logR*) be the green and red background-corrected log intensities. Then, (*M*, *A*) are defined by *M *= *log*(*R/G*) and . Note that M is the unnormalized log ratio.

The adjusted log ratio for the jth miR is computed by: M_j_*(A_j_) = M_j _- c(A_j_), where c(A_j_) is the lowess curve fit to the MA plot. For the calculations presented in this paper, the lowess curve was calculated using the R function loess with a span set at 0.5 [16].

5) Quantile-quantile

Quantile normalization [17] assumes that the distribution of miR abundances is nearly the same in all samples. For convenience, an artificial reference chip is created by pooling intensities across all chips in the experiment to produce an intensity reference distribution. This reference distribution is described by a distribution function *F_2_*. To normalize each chip, the distribution of miR intensities for that chip (e.g. denoted by the distribution function *F_1_*) is transformed to equal the reference intensity distribution. Operationally, this transformation is accomplished by determining for each signal intensity on the chip its quantile in the chip's intensity distribution and replacing that value with the value having that quantile in the reference distribution. In a formula, the transform is

*x_norm _= F_2_^-1^(F_1_(x))*,

where *F_1 _*is the distribution function of the actual chip, and *F_2 _*is the distribution function of the reference chip.

6) Invariant set option

Sometimes the normalization factors or curves calculated as described above are derived using only an invariant subset of the probes (e.g., miRs). The notion of invariant set normalization was first introduced for Affymetrix gene expression chips [18], but it can be generalized to miR arrays. This method assumes that there is a set of reference miRs that are invariant across a set of samples. Rather than requiring *a priori *specification of a standard set of "housekeeping miRs", the invariant set is determined empirically. The invariant probes are identified by determining those probes which have most similar rank order across all arrays as measured by the smallest variance of ranks. There is some arbitrariness in deciding what percentage of the probes belong in the invariant set, so in our study we considered several possible percentages, including 10%, 20%, 30% and 40% of the probes with the smallest variance to serve as the "invariant set". Normalization methods 1) to 5) were then reapplied based on the defined invariant sets of miRs. The invariant set of miRs including 40% of the probes with smallest variance was used only for the quantile normalization method.

The shorthand notation used to indicate the various normalization methods is the name of the main approach (Median, Mean, trimmed Mean, Lowess, or Quantile) with a suffix indicating the size of the invariant set used, if any (.10,.20,.30,.40). No suffix indicates that the full set of miRs was used.

### Measures of variation

We examined the impact of each normalization method on both within miR error variance (between replicate arrays) and between miR variance. This analysis was based on a components of variance model:

where *Y*_*ij *_denotes the log transformed intensity ratio of *i*th miR in the *j*th replicate. The error variance component  associated with *e*_*ij *_(technical error) represents the reproducibility of the method. The variance component  associated with *m_i _*(true miR expression) represents the true miR-to-miR variability. Formulas for computing the variance components and intra-class correlation based on method-of-moments estimation for each cell line under each normalization method can be computed as in Korn et al. [19]. The error variance (within-miR) variance component is estimated by

where *n_a _*= number of replicate arrays, *n_m _*= number of miRs and

The between-miR variance component is estimated by

The estimated intra-class correlation (*ICC*) for each cell line is

and it estimates the proportion of the total variance (sum of within and between miR variances) due to the between miR variance. It is desirable for the *ICC *to be large (close to one), indicating that the technical error variance is relatively small compared to biological differences between miRs [19]. When the error variance is fairly high, it is possible for the estimated *ICC *to be negative due to use of method-of-moments estimation, especially when the number of technical replicates is small. The advantage of the method-of-moments estimators is that they are unbiased and simple to compute.

### Statistical tests for differences in ICC between normalization methods

We examined the following normalization methods: no normalization, mean, median, trimmed mean, lowess and quantile normalization based on all miRs (N = 6 normalization methods); based on the three invariant sets defined above for the mean, median, trimmed mean, and lowess methods (N = 12); and based on four invariant sets for the quantile method (N = 4). For each of these normalization methods, there were 19 *ICC *values computed, corresponding to 10 lung cancer cell lines and 9 renal cancer cell lines. Separately for the lung cancer cell lines and the renal cancer cell lines, Wilcoxon signed-rank tests were applied to the ICC for each of the 231 possible pairings of these methods. Two methods were considered statistically significantly different if the 2-sided p-value from the signed-rank test was less than α = 0.01. This α level was chosen so that the expected number of false positive differences would be no more than 3 among the 231 paired tests for each of the two cell line experiments.

## Results

**Figure 1:**
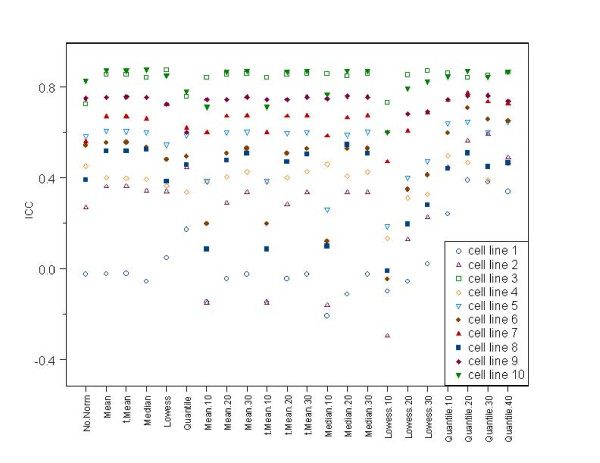
**Dot plot for comparison of *ICC*s observed for different normalization methods applied to replicate miR microarray data from 10 lung cancer cell lines**. The y axis is the intra-class correlation coefficient (*ICC*), and the x-axis lists different normalization methods. The x-axis indicates the normalization method used. The shorthand notation for the normalization method is the name of the main approach (Median, Mean, trimmed Mean, Lowess, or Quantile) with a suffix indicating the size of the invariant set used, if any (.10,.20,.30,.40). No suffix indicates that the full set of miRs was used.

The *ICC*s for different normalization methods using the ten lung cancer cell lines ranged from -0.30 to 0.87 (see Table 1, 2 and Figure 1). The quantile normalization methods based on invariant sets were observed to produce the highest mean *ICCs *across the ten lung cancer cell lines (mean *ICC *> 0.60, for all invariant set sizes 10-40%). The worst performing methods were the lowess methods when based on invariant sets (mean *ICC *< 0.50). For all pairwise comparisons of invariant set quantile normalization versus invariant set lowess normalization, the distribution of *ICC*s was significantly lower for the lowess-based methods compared to the quantile-based methods (P < 0.01 for all pairs, Wilcoxon signed rank tests). Cell line effects were also apparent, with the lowest average *ICC *observed for cell line 1 (mean *ICC *= 0.02, empty blue circle in Figure 1) and the highest average *ICC *observed for cell line 3 (mean *ICC *= 0.84, empty green square in Figure 1). When using the full data set (not restricting to an invariant set), global mean, global trimmed-mean, and global median performed about equally well, although those *ICC*s were somewhat lower than the *ICC*s for the quantile-based methods using invariant sets. With the exception of the lowess methods and methods using small invariant sets (e.g., 10%), performing some type of normalization generally produced higher *ICC*s than performing no normalization.

**Table 1:** Summary statistics for performance of different normalization methods based on intra-class correlations (*ICC*s) computed for replicate miR microarray data obtained using 10 different lung cancer cell lines

Methods	Min	Max	Median	Mean	SD
No.Norm	-0.03	0.82	0.55	0.51	0.25
Mean	-0.02	0.87	0.58	0.56	0.27
t.Mean	-0.02	0.87	0.58	0.56	0.27
Median	-0.06	0.87	0.56	0.54	0.27
Lowess	0.05	0.87	0.51	0.53	0.26
Quantile	0.17	0.78	0.54	0.52	0.18
Mean.10	-0.15	0.84	0.38	0.36	0.36
Mean.20	-0.05	0.86	0.55	0.54	0.28
Mean.30	-0.03	0.87	0.56	0.55	0.27
t.Mean.10	-0.15	0.84	0.38	0.36	0.36
t.Mean.20	-0.05	0.86	0.55	0.53	0.28
t.Mean.30	-0.02	0.87	0.56	0.55	0.27
Median.10	-0.21	0.86	0.36	0.35	0.39
Median.20	-0.11	0.87	0.56	0.54	0.29
Median.30	-0.07	0.87	0.57	0.55	0.28
Lowess.10	-0.30	0.73	0.16	0.23	0.35
Lowess.20	-0.06	0.85	0.37	0.42	0.30
Lowess.30	0.02	0.87	0.44	0.48	0.28
Quantile.10	0.24	0.86	0.62	0.60	0.20
Quantile.20	0.39	0.87	0.67	0.65	0.16
Quantile.30	0.38	0.85	0.63	0.62	0.18
Quantile.40	0.34	0.86	0.65	0.62	0.18

**Table 2:** Summary statistics for 10 different lung cancer cell lines based on intra-class correlations (ICCs) computed for replicate miR microarray data processed using different normalization methods

Cell lines	Min	Max	Median	Mean	SD
1	-0.21	0.39	-0.03	0.02	0.17
2	-0.30	0.59	0.33	0.26	0.24
3	0.72	0.87	0.85	0.84	0.04
4	0.13	0.50	0.40	0.39	0.07
5	0.18	0.64	0.59	0.53	0.13
6	-0.05	0.71	0.53	0.46	0.19
7	0.47	0.77	0.67	0.66	0.07
8	-0.01	0.55	0.46	0.38	0.18
9	0.60	0.76	0.74	0.73	0.05
10	0.60	0.87	0.85	0.82	0.07

**Figure 2:**
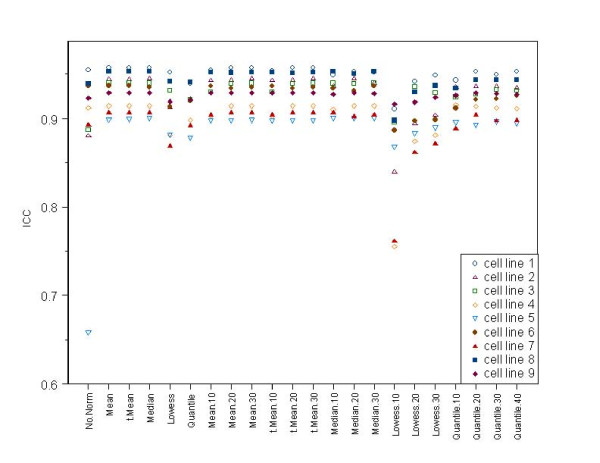
**Dot plot for comparison of *ICC*s observed for different normalization methods applied to replicate miR microarray data from 9 renal cancer cell lines**. The y axis is the intra-class correlation coefficient (*ICC*), and the x-axis lists different normalization methods. The x-axis indicates the normalization method used. The shorthand notation for the normalization method is the name of the main approach (Median, Mean, trimmed Mean, Lowess, or Quantile) with a suffix indicating the size of the invariant set used, if any (.10,.20,.30,.40). No suffix indicates that the full set of miRs was used.

The *ICC*s for different normalization methods for the experiment involving nine renal cancer cell lines ranged from 0.66 to 0.96 (see Table 3, 4 and Figure 2). Overall, the *ICC*s were higher for the renal cell lines than for the lung cancer cell lines, likely due to the more controlled setting in which the renal cancer cell lines were processed, although it is possible that biological differences between the lung and renal cell lines could also partly explain the findings. The entire set of renal cancer cell line experiments was performed in one flash page batch by one technician, in contrast to the lung cancer cell line experiments, which were processed in several batches. When using the full set of miRs for normalization, the mean, trimmed mean, and median normalization methods all produced similarly high *ICC*s. As was observed for the lung cancer cell line experiments, the lowess methods based on invariant sets tended to produce lower *ICC*s and the quantile methods based on invariant sets tended to produce higher *ICC*s. Comparing invariant set quantile normalization to invariant set lowess normalization, *ICC*s were always observed to be lower for the lowess-based methods compared to the quantile-based methods with the pairwise differences reaching statistical significance for most pairs (P < 0.01 for most pairs, Wilcoxon signed rank tests) [Additional file 1, 2]. With the exception of the lowess method based on 10% invariant set, performing some type of normalization produced a higher *ICC *than performing no normalization at all.

**Table 3:** Summary statistics for performance of different normalization methods based on intra-class correlations (*ICC*s) computed for replicate miR microarray data obtained using 9 different renal cancer cell lines

Methods	Min	Max	Median	Mean	SD
No.Norm	0.66	0.95	0.91	0.89	0.09
Mean	0.90	0.96	0.94	0.93	0.02
t.Mean	0.90	0.96	0.94	0.93	0.02
Median	0.90	0.96	0.94	0.93	0.02
Lowess	0.87	0.95	0.91	0.91	0.03
Quantile	0.88	0.94	0.92	0.91	0.02
Mean.10	0.90	0.95	0.93	0.93	0.02
Mean.20	0.90	0.96	0.93	0.93	0.02
Mean.30	0.90	0.96	0.94	0.93	0.02
t.Mean.10	0.90	0.95	0.93	0.93	0.02
t.Mean.20	0.90	0.96	0.93	0.93	0.02
t.Mean.30	0.90	0.96	0.94	0.93	0.02
Median.10	0.90	0.95	0.93	0.93	0.02
Median.20	0.90	0.95	0.93	0.93	0.02
Median.30	0.90	0.95	0.94	0.93	0.02
Lowess.10	0.75	0.92	0.89	0.86	0.06
Lowess.20	0.86	0.94	0.90	0.90	0.03
Lowess.30	0.87	0.95	0.90	0.91	0.03
Quantile.10	0.89	0.94	0.92	0.92	0.02
Quantile.20	0.89	0.95	0.93	0.92	0.02
Quantile.30	0.90	0.95	0.93	0.92	0.02
Quantile.40	0.89	0.95	0.93	0.92	0.02

**Table 4:** Summary statistics for 9 different renal cancer cell lines based on intra-class correlations (ICCs) computed for replicate miR microarray data processed using different normalization methods

Cell lines	Min	Max	Median	Mean	SD
1	0.91	0.96	0.95	0.95	0.01
2	0.84	0.95	0.94	0.93	0.03
3	0.89	0.94	0.93	0.93	0.01
4	0.75	0.92	0.91	0.90	0.03
5	0.66	0.90	0.90	0.88	0.05
6	0.89	0.94	0.93	0.93	0.02
7	0.76	0.91	0.90	0.89	0.03
8	0.90	0.95	0.95	0.94	0.01
9	0.92	0.93	0.93	0.93	0.00

## Discussion

Data normalization is an important step in the analysis of microarray data. We explored a comprehensive collection of normalization methods in miR microarray experiments using lung cancer cell lines and renal cancer cell lines to address the question of which normalization methods might be most appropriate for miR microarray data. We tested global mean, trimmed mean, global median, lowess, and quantile-quantile methods and examined the impact of using each of these methods restricted to an empirically determined invariant miR set. We found that for our data sets, lowess normalization generally did not perform as well as the other methods. For the lung cancer cell lines quantile normalization applied to an invariant set was best on average unless restricted to a very small invariant set (e.g., 10%). Quantile normalization with invariant set also performed well for the renal cancer cell lines, but average observed ICCs were slightly higher for global median and mean methods. The good performance of quantile normalization restricted to an invariant miR set observed in our study is consistent with a previous study reported for a one channel miR chip [11]. Global median and global mean methods performed reasonably well in both data sets and have the advantage of computational simplicity.

Although many different normalization methods have been used for gene expression microarray data, there may be characteristics of miR expression that will influence the optimal choice of normalization method for miR microarray data. The number of probes on a miR microarray is typically much smaller (a few hundred or less) than the number of probes on a gene expression cDNA microarray (usually tens of thousands), and the expected proportion of differentially expressed miRs comparing across samples in a miR microarray experiment might be higher than the proportion of differentially expressed genes typically expected for gene expression microarray studies. It may be difficult to anticipate what percentage of miRs are likely to be truly invariant across a set of samples used in an experiment, so ad hoc decisions may have to be made for the invariant set size to be used for normalization methods that use invariant sets. Our results suggested that using an invariant set consisting of only 10% of the miRs resulted in diminished performance compared to methods using larger invariant sets, but the appropriate invariant set size obviously could depend on the particular experimental setting. Global mean and median methods require assumptions that either the number of differentially expressed miRs is not too large or that the amount of over-expression and under-expression of miRs within each sample is somehow balanced so that the mean or median is still a reasonable indicator of overall shift in expression level due to technical factors. Researchers still need to consider carefully which assumptions underlying the different normalization methods appear most reasonable for their experimental setting and possibly consider more than one normalization approach to determine the sensitivity of their results to normalization method used.

## Competing interests

The authors declare that they have no competing interests.

## Authors' contributions

YZ, EW, MTL, and LMM conceived of the study. YZ and LMM proposed the experimental design with input from EW, MTL, FMM, MR, and JK. EW and LH performed the miR array experiments. YZ performed the statistical analyses with input from LMM. YZ, EW, and LMM drafted the manuscript. All authors read and approved the final version of the manuscript.

## Supplementary Material

Additional file 1 **Table presenting p-values resulting from Wilcoxon signed-rank tests used to compare *ICC*s of different normalization methods applied to data obtained by miR microarray analysis of 10 lung cancer cell lines**. Click here for file

Additional file 2 **Table presenting p-values resulting from Wilcoxon signed-rank tests used to compare *ICC*s of different normalization methods applied to data obtained by miR microarray analysis of 9 renal cancer cell lines**. Click here for file
